# 16-Oxa­penta­cyclo­[6.6.5.0^1,18^.0^2,7^.0^9,14^]nona­deca-2,4,6,9,11,13,18-heptaen-15-one

**DOI:** 10.1107/S1600536814000026

**Published:** 2014-01-08

**Authors:** Eason M. Mathew, M. Sithambaresan, P. A. Unnikrishnan, M. R. Prathapachandra Kurup

**Affiliations:** a Department of Applied Chemistry, Cochin University of Science and Technology, Kochi 682022, India; b Department of Chemistry, Faculty of Science, Eastern University, Chenkalady, Sri Lanka

## Abstract

In the title compound, C_18_H_12_O_2_, the benzene rings are inclined to one another by 66.79 (7)°. The five-membered ring is almost planar with a maximum deviation of 0.014 (1) Å. In the crystal, the mol­ecules are linked by pairs of weak C—H⋯O interactions into centrosymmetric dimers. These dimers are linked by C—H⋯π interactions, forming a three-dimensional structure.

## Related literature   

For background to dibenzobarrelene dervatives and their applications, see: Khalil *et al.* (2010 ▶); Cox *et al.* (2013 ▶). For the synthesis of related compounds, see: Ciganek (1980 ▶); De Luca *et al.* (2001 ▶). For a related structure, see: Mathew *et al.* (2013 ▶). For puckering analysis, see: Cremer & Pople (1975 ▶).
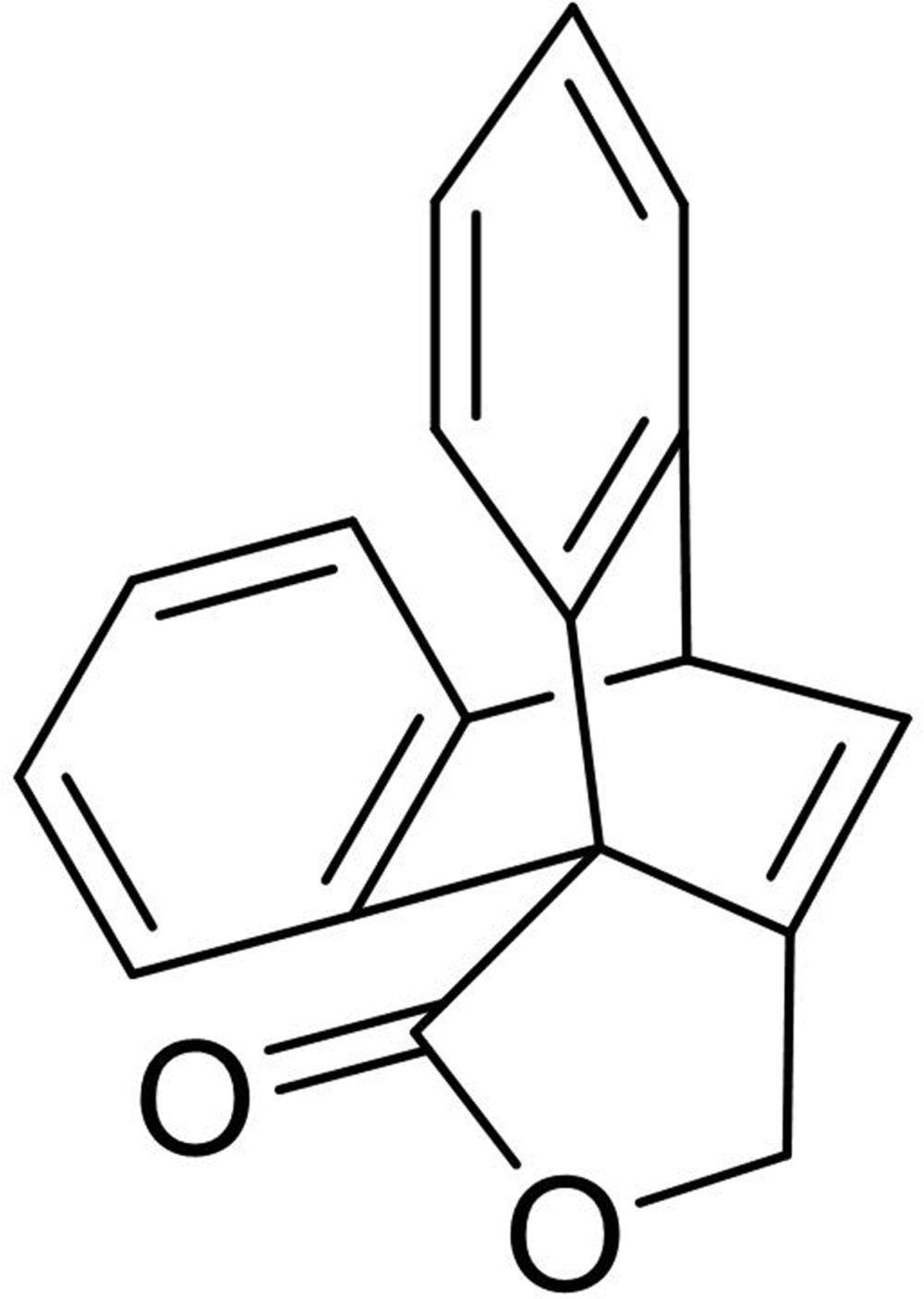


## Experimental   

### 

#### Crystal data  

C_18_H_12_O_2_
*M* *_r_* = 260.28
Monoclinic, 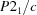

*a* = 9.351 (1) Å
*b* = 13.8153 (16) Å
*c* = 10.1514 (9) Å
β = 105.295 (4)°
*V* = 1265.0 (2) Å^3^
*Z* = 4
Mo *K*α radiation
μ = 0.09 mm^−1^
*T* = 296 K
0.40 × 0.35 × 0.30 mm

#### Data collection  

Bruker Kappa APEXII CCD area-detector diffractometer
Absorption correction: multi-scan (*SADABS*; Bruker, 2007 ▶) *T*_min_ = 0.966, *T*_max_ = 0.974
9570 measured reflections
3123 independent reflections
2224 reflections with *I* > 2σ(*I*)
*R*_int_ = 0.035

#### Refinement  

*R*[*F* ^2^ > 2σ(*F* ^2^)] = 0.044
*wR*(*F* ^2^) = 0.132
*S* = 1.03
3123 reflections
182 parameters
H-atom parameters constrained
Δρ_max_ = 0.22 e Å^−3^
Δρ_min_ = −0.19 e Å^−3^

### 

Data collection: *APEX2* (Bruker, 2007 ▶); cell refinement: *SAINT* (Bruker, 2007 ▶); data reduction: *SAINT*; program(s) used to solve structure: *SHELXS97* (Sheldrick, 2008 ▶); program(s) used to refine structure: *SHELXL97* (Sheldrick, 2008 ▶); molecular graphics: *ORTEP-3 for Windows* (Farrugia, 2012 ▶) and *DIAMOND* (Brandenburg, 2010 ▶); software used to prepare material for publication: *SHELXL97* and *publCIF* (Westrip, 2010 ▶).

## Supplementary Material

Crystal structure: contains datablock(s) I, Global. DOI: 10.1107/S1600536814000026/yk2102sup1.cif

Structure factors: contains datablock(s) I. DOI: 10.1107/S1600536814000026/yk2102Isup2.hkl

Supporting information file. DOI: 10.1107/S1600536814000026/yk2102Isup3.cml

CCDC reference:

Additional supporting information:  crystallographic information; 3D view; checkCIF report

## Figures and Tables

**Table 1:** Hydrogen-bond geometry (Å, °) *Cg*1 and *Cg*2 are the centroids of the C1–C6 and C8–C13 rings, respectively.

*D*—H⋯*A*	*D*—H	H⋯*A*	*D*⋯*A*	*D*—H⋯*A*
C7—H7⋯*Cg*1^i^	0.98	2.92	3.7835 (16)	148
C16—H16*B*⋯*Cg*1^ii^	0.97	2.76	3.650 (2)	153
C18—H18⋯*Cg*2^i^	0.93	2.84	3.3942 (16)	120
C3—H3⋯O1^iii^	0.93	2.64	3.519 (2)	157

Symmetry codes: (i) 
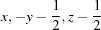
; (ii) 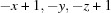; (iii) 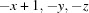.

**Fig. 1.**
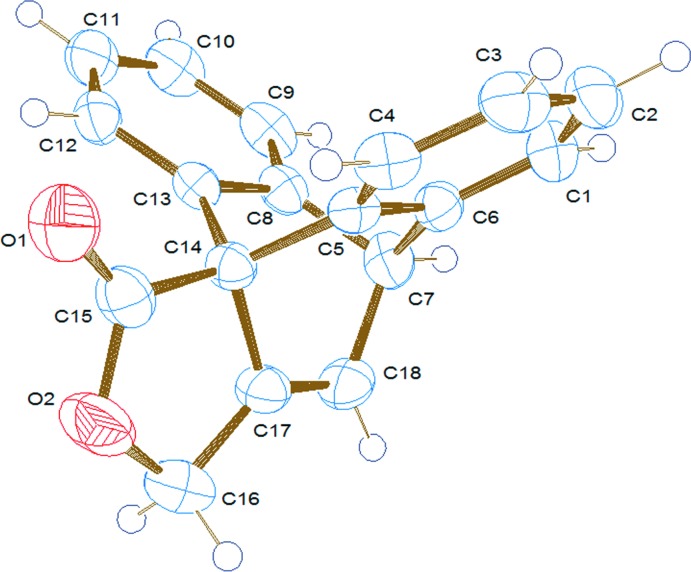
ORTEP view of the title molecule drawn with 50% probability displacement ellipsoids and showing the atom labelling scheme.

**Fig. 2.**
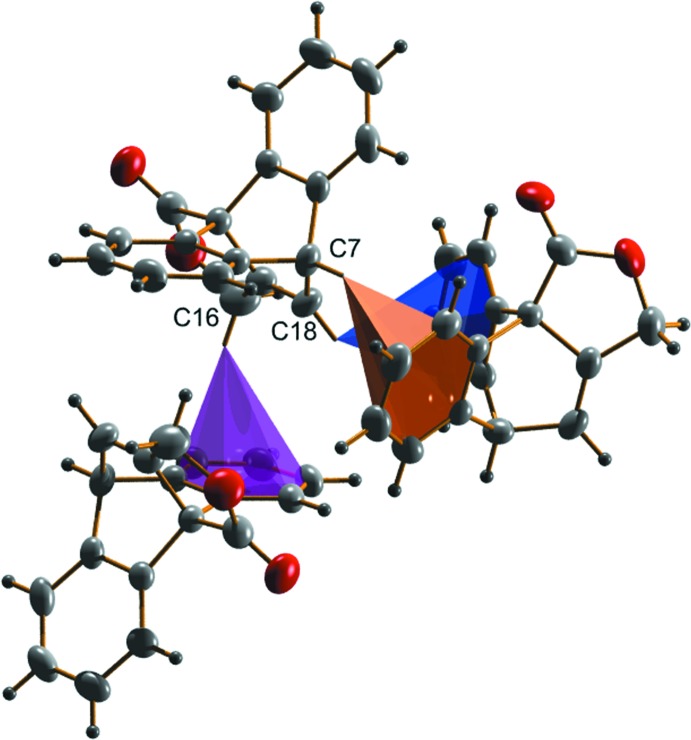
C—H···π interactions in the title compound.

**Fig. 3.**
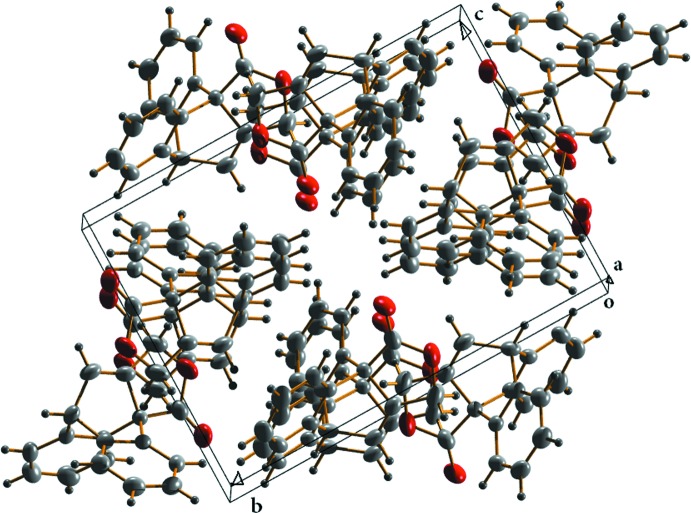
Packing diagram of the compound along a axis.

## supplementary crystallographic information

### 1. Comment

Dibenzobarrelene derivatives have been used as the key intermediates in the synthesis of several compounds which show various biological activities. Besides, they have showed application in drug development for various diseases (Khalil *et al.*, 2010) and in the alignment of nematic liquid crystals (Cox *et al.*, 2013). As a continuous work on the dibenzobarrelene compounds, a new dibenzobarrelene derivative, 3,5-dihydro-5,9 b-*o*-benzonaphtho [1,2-*c*] furan-1-one, was prepared and structurally characterized. The *ORTEP* view of the title compound is shown in Fig. 1.

The compound crystallizes in monoclinic space group *P*2_1_/*c*. The dihedral angle of the two aromatic rings of the anthracene moiety is 66.79 (7)°. The five-membered heterocyclic ring C14–17/O2 is almost planar with a maximum deviation of 0.014 (1)° for C14 carbon atom in the ring. One of the fused six-membered ring of the molecule C5—C8/C13/C14 is in boat conformation [φ = 117.52 (10)° and θ = 90.62 (10)°] having total puckering amplitude Q_T_ of 0.8495 (15) Å. The other six-membered ring C5—C7/C14/C17/C18 is also in boat conformation [φ = 302.45 (11)° and θ = 89.63 (12)°] having total puckering amplitude Q_T_ of 0.7919 (15) Å. Another six-membered ring C7/C8/C13/C14/C17/C18 is also in the same conformation [φ = 181.09 (11)° and θ = 90.35 (11)°] having total puckering amplitude Q_T_ of 0.7915 (15) Å (Cremer & Pople, 1975).

Three C–H···π interactions (Fig. 2) are found in the molecule. Whilst the two C–H···π interactions exist between the H atoms attached at the C7 and C16 atoms and the aromatic rings (C1—C6) of the anthracene moiety of two adjacent molecules from opposite sides of the main molecule, the third C–H···π interaction exists between the hydrogen at C18 atom and the C8—C13 ring of one of the above neighbouring molecules respectively with H···π distances of 2.92, 2.76 and 2.84 Å (Table 1). There are very weak π···π interactions between the aromatic rings of the anthracene moiety with centroid-centroid distances greater than 4 Å. However, packing of molecules is predominantly favored by the C–H···π interactions. Similar intermolecular interactive behaviour was found in the compound 8-phenyl-16-thiapentacyclo[6.6.5.01,18.02,7.09,14]nonadeca-2,4,6,9,11,13,18- heptane (Mathew *et al.*, 2013). Fig. 3 shows the packing diagram of the title compound along *a* axis.

### 2. Experimental

The title compound was prepared by adapting a reported procedure (Ciganek, 1980; De Luca *et al.*, 2001). Triethylamine (0.70 ml, 5 mmol) was added to a solution of 9-anthracene carboxylic acid (1.11 g, 5 mmol) and cyanuric chloride (0.92 g, 5 mmol) in acetone and stirred at room temperature for 1 h to obtain 9-anthracene acid chloride. Propargyl alcohol (0.30 ml, 5 mmol) was added to it and the mixture was stirred for 4 h. Propargyl-9-anthroate obtained was purified by silica gel column chromatography using a mixture of hexane and dichloromethane as eluents. Propargyl-9-anthroate was refluxed in 10 ml of *p*-xylene for 48 h (intramolecular Diels-Alder reaction) to generate the title compound. Colourless crystals acceptable for X-ray structure determination were recrystallized from acetonitrile by slow evaporation over a few days (m.p: 260 °C).

### 3. Refinement

All H atoms on C were placed in calculated positions, guided by difference maps, with C–H bond distances 0.93–0.97 Å. H atoms were assigned as *U*_iso_=1.2Ueq. Omitted owing to bad disagreement were the reflections (1 0 0).

### Crystal data

**Table:**

C_18_H_12_O_2_	*F*(000) = 544
*M**_r_* = 260.28	*D*_x_ = 1.367 Mg m^−^^3^
Monoclinic, *P*2_1_/*c*	Mo *K*α radiation, λ = 0.71073 Å
Hall symbol: -P 2ybc	Cell parameters from 2538 reflections
*a* = 9.351 (1) Å	θ = 2.7–27.2°
*b* = 13.8153 (16) Å	µ = 0.09 mm^−^^1^
*c* = 10.1514 (9) Å	*T* = 296 K
β = 105.295 (4)°	Block, colourless
*V* = 1265.0 (2) Å^3^	0.40 × 0.35 × 0.30 mm
*Z* = 4	

### Data collection

**Table:**

Bruker Kappa APEXII CCD area-detector diffractometer	3123 independent reflections
Radiation source: fine-focus sealed tube	2224 reflections with *I* > 2σ(*I*)
Graphite monochromator	*R*_int_ = 0.035
Detector resolution: 8.33 pixels mm^-1^	θ_max_ = 28.3°, θ_min_ = 2.7°
ω and φ scan	*h* = −12→12
Absorption correction: multi-scan (*SADABS*; Bruker, 2007)	*k* = −18→18
*T*_min_ = 0.966, *T*_max_ = 0.974	*l* = −9→13
9570 measured reflections	

### Refinement

**Table:**

Refinement on *F*^2^	Secondary atom site location: difference Fourier map
Least-squares matrix: full	Hydrogen site location: inferred from neighbouring sites
*R*[*F*^2^ > 2σ(*F*^2^)] = 0.044	H-atom parameters constrained
*wR*(*F*^2^) = 0.132	*w* = 1/[σ^2^(*F*_o_^2^) + (0.0716*P*)^2^ + 0.0884*P*] where *P* = (*F*_o_^2^ + 2*F*_c_^2^)/3
*S* = 1.03	(Δ/σ)_max_ < 0.001
3123 reflections	Δρ_max_ = 0.22 e Å^−^^3^
182 parameters	Δρ_min_ = −0.19 e Å^−^^3^
0 restraints	Extinction correction: *SHELXL*, Fc^*^=kFc[1+0.001xFc^2^λ^3^/sin(2θ)]^-1/4^
Primary atom site location: structure-invariant direct methods	Extinction coefficient: 0.042 (4)

### Special details

**Table:**

Geometry. All e.s.d.'s (except the e.s.d. in the dihedral angle between two l.s. planes) are estimated using the full covariance matrix. The cell e.s.d.'s are taken into account individually in the estimation of e.s.d.'s in distances, angles and torsion angles; correlations between e.s.d.'s in cell parameters are only used when they are defined by crystal symmetry. An approximate (isotropic) treatment of cell e.s.d.'s is used for estimating e.s.d.'s involving l.s. planes.
Refinement. Refinement of *F*^2^ against ALL reflections. The weighted *R*-factor *wR* and goodness of fit *S* are based on *F*^2^, conventional *R*-factors *R* are based on *F*, with *F* set to zero for negative *F*^2^. The threshold expression of *F*^2^ > σ(*F*^2^) is used only for calculating *R*-factors(gt) *etc*. and is not relevant to the choice of reflections for refinement. *R*-factors based on *F*^2^ are statistically about twice as large as those based on *F*, and *R*- factors based on ALL data will be even larger.

### Fractional atomic coordinates and isotropic or equivalent isotropic displacement parameters (Å^2^)

**Table:**

	*x*	*y*	*z*	*U*_iso_*/*U*_eq_	
O1	0.80024 (14)	−0.01503 (9)	0.22190 (12)	0.0570 (4)	
O2	0.81229 (15)	−0.05665 (8)	0.43556 (13)	0.0590 (4)	
C1	0.35542 (16)	0.23405 (12)	0.28342 (15)	0.0410 (4)	
H1	0.3188	0.2869	0.3209	0.049*	
C2	0.27224 (16)	0.19194 (12)	0.16350 (15)	0.0454 (4)	
H2	0.1799	0.2175	0.1198	0.054*	
C3	0.32484 (18)	0.11291 (12)	0.10856 (15)	0.0440 (4)	
H3	0.2663	0.0840	0.0300	0.053*	
C4	0.46477 (17)	0.07589 (11)	0.16959 (13)	0.0368 (3)	
H4	0.5010	0.0230	0.1318	0.044*	
C5	0.54912 (15)	0.11890 (10)	0.28707 (13)	0.0294 (3)	
C6	0.49305 (15)	0.19640 (10)	0.34614 (13)	0.0323 (3)	
C7	0.59990 (16)	0.23295 (11)	0.47731 (13)	0.0372 (4)	
H7	0.5584	0.2858	0.5201	0.045*	
C8	0.73588 (15)	0.26293 (10)	0.43345 (13)	0.0329 (3)	
C9	0.79945 (18)	0.35413 (11)	0.44610 (15)	0.0426 (4)	
H9	0.7627	0.4033	0.4905	0.051*	
C10	0.91836 (18)	0.37120 (12)	0.39195 (16)	0.0472 (4)	
H10	0.9615	0.4323	0.4001	0.057*	
C11	0.97316 (17)	0.29906 (12)	0.32652 (16)	0.0462 (4)	
H11	1.0519	0.3120	0.2893	0.055*	
C12	0.91213 (15)	0.20649 (11)	0.31525 (14)	0.0373 (3)	
H12	0.9503	0.1573	0.2720	0.045*	
C13	0.79444 (14)	0.18928 (10)	0.36931 (12)	0.0297 (3)	
C14	0.70748 (15)	0.09493 (10)	0.36761 (13)	0.0298 (3)	
C15	0.77607 (17)	0.00483 (11)	0.32910 (16)	0.0408 (4)	
C16	0.7720 (2)	−0.01959 (13)	0.55478 (17)	0.0566 (5)	
H16A	0.8592	−0.0126	0.6311	0.068*	
H16B	0.7028	−0.0629	0.5813	0.068*	
C17	0.70234 (16)	0.07596 (12)	0.51340 (14)	0.0375 (3)	
C18	0.64493 (17)	0.14591 (12)	0.57090 (14)	0.0419 (4)	
H18	0.6331	0.1426	0.6589	0.050*	

### Atomic displacement parameters (Å^2^)

**Table:**

	*U*^11^	*U*^22^	*U*^33^	*U*^12^	*U*^13^	*U*^23^
O1	0.0749 (9)	0.0426 (7)	0.0586 (7)	0.0140 (6)	0.0266 (6)	−0.0097 (6)
O2	0.0767 (9)	0.0353 (7)	0.0645 (8)	0.0168 (6)	0.0177 (6)	0.0126 (6)
C1	0.0341 (8)	0.0432 (9)	0.0454 (8)	0.0049 (6)	0.0097 (6)	−0.0012 (7)
C2	0.0322 (8)	0.0528 (10)	0.0457 (8)	−0.0005 (7)	0.0005 (7)	0.0072 (7)
C3	0.0429 (8)	0.0487 (10)	0.0339 (7)	−0.0133 (7)	−0.0015 (6)	0.0007 (7)
C4	0.0452 (8)	0.0319 (7)	0.0332 (7)	−0.0058 (6)	0.0099 (6)	−0.0028 (6)
C5	0.0327 (7)	0.0288 (7)	0.0268 (6)	−0.0021 (5)	0.0079 (5)	0.0025 (5)
C6	0.0311 (7)	0.0355 (8)	0.0304 (6)	−0.0006 (5)	0.0085 (5)	0.0000 (6)
C7	0.0383 (8)	0.0412 (8)	0.0311 (7)	0.0048 (6)	0.0076 (6)	−0.0096 (6)
C8	0.0330 (7)	0.0337 (7)	0.0276 (6)	0.0023 (6)	0.0003 (5)	−0.0045 (5)
C9	0.0451 (9)	0.0339 (8)	0.0396 (8)	0.0026 (6)	−0.0049 (7)	−0.0080 (6)
C10	0.0407 (9)	0.0386 (9)	0.0532 (9)	−0.0103 (7)	−0.0035 (7)	0.0001 (7)
C11	0.0296 (7)	0.0513 (10)	0.0542 (9)	−0.0062 (6)	0.0051 (7)	0.0080 (8)
C12	0.0292 (7)	0.0400 (8)	0.0417 (7)	0.0033 (6)	0.0073 (6)	0.0026 (6)
C13	0.0295 (7)	0.0289 (7)	0.0279 (6)	0.0029 (5)	0.0026 (5)	0.0007 (5)
C14	0.0337 (7)	0.0271 (7)	0.0286 (6)	0.0017 (5)	0.0080 (5)	0.0002 (5)
C15	0.0437 (8)	0.0306 (8)	0.0477 (8)	0.0037 (6)	0.0111 (7)	−0.0007 (7)
C16	0.0646 (11)	0.0535 (11)	0.0507 (9)	0.0097 (9)	0.0136 (9)	0.0200 (8)
C17	0.0371 (8)	0.0429 (8)	0.0307 (7)	0.0001 (6)	0.0056 (6)	0.0074 (6)
C18	0.0426 (8)	0.0582 (10)	0.0251 (6)	−0.0012 (7)	0.0092 (6)	0.0013 (6)

### Geometric parameters (Å, º)

**Table:**

O1—C15	1.2001 (19)	C8—C9	1.385 (2)
O2—C15	1.3457 (19)	C8—C13	1.3955 (19)
O2—C16	1.452 (2)	C9—C10	1.384 (2)
C1—C6	1.3783 (19)	C9—H9	0.9300
C1—C2	1.388 (2)	C10—C11	1.370 (2)
C1—H1	0.9300	C10—H10	0.9300
C2—C3	1.375 (2)	C11—C12	1.393 (2)
C2—H2	0.9300	C11—H11	0.9300
C3—C4	1.389 (2)	C12—C13	1.374 (2)
C3—H3	0.9300	C12—H12	0.9300
C4—C5	1.3788 (19)	C13—C14	1.5339 (19)
C4—H4	0.9300	C14—C15	1.499 (2)
C5—C6	1.395 (2)	C14—C17	1.5163 (18)
C5—C14	1.5272 (18)	C16—C17	1.483 (2)
C6—C7	1.5246 (18)	C16—H16A	0.9700
C7—C8	1.512 (2)	C16—H16B	0.9700
C7—C18	1.521 (2)	C17—C18	1.315 (2)
C7—H7	0.9800	C18—H18	0.9300
			
C15—O2—C16	112.40 (12)	C11—C10—H10	119.6
C6—C1—C2	119.08 (15)	C9—C10—H10	119.6
C6—C1—H1	120.5	C10—C11—C12	120.68 (15)
C2—C1—H1	120.5	C10—C11—H11	119.7
C3—C2—C1	120.74 (14)	C12—C11—H11	119.7
C3—C2—H2	119.6	C13—C12—C11	118.69 (14)
C1—C2—H2	119.6	C13—C12—H12	120.7
C2—C3—C4	120.47 (13)	C11—C12—H12	120.7
C2—C3—H3	119.8	C12—C13—C8	120.96 (13)
C4—C3—H3	119.8	C12—C13—C14	128.37 (12)
C5—C4—C3	118.93 (14)	C8—C13—C14	110.66 (12)
C5—C4—H4	120.5	C15—C14—C17	103.71 (12)
C3—C4—H4	120.5	C15—C14—C5	117.50 (11)
C4—C5—C6	120.55 (13)	C17—C14—C5	106.50 (11)
C4—C5—C14	128.48 (13)	C15—C14—C13	116.53 (12)
C6—C5—C14	110.95 (11)	C17—C14—C13	106.81 (11)
C1—C6—C5	120.13 (13)	C5—C14—C13	104.96 (10)
C1—C6—C7	126.55 (14)	O1—C15—O2	121.07 (14)
C5—C6—C7	113.29 (11)	O1—C15—C14	128.49 (14)
C8—C7—C18	106.55 (12)	O2—C15—C14	110.44 (13)
C8—C7—C6	104.00 (11)	O2—C16—C17	105.60 (12)
C18—C7—C6	107.06 (12)	O2—C16—H16A	110.6
C8—C7—H7	112.9	C17—C16—H16A	110.6
C18—C7—H7	112.9	O2—C16—H16B	110.6
C6—C7—H7	112.9	C17—C16—H16B	110.6
C9—C8—C13	119.68 (14)	H16A—C16—H16B	108.8
C9—C8—C7	126.58 (14)	C18—C17—C16	136.81 (14)
C13—C8—C7	113.68 (12)	C18—C17—C14	115.35 (13)
C10—C9—C8	119.23 (14)	C16—C17—C14	107.80 (13)
C10—C9—H9	120.4	C17—C18—C7	112.45 (12)
C8—C9—H9	120.4	C17—C18—H18	123.8
C11—C10—C9	120.73 (15)	C7—C18—H18	123.8
			
C6—C1—C2—C3	0.9 (2)	C4—C5—C14—C17	−125.40 (15)
C1—C2—C3—C4	−2.3 (2)	C6—C5—C14—C17	56.01 (15)
C2—C3—C4—C5	0.8 (2)	C4—C5—C14—C13	121.57 (15)
C3—C4—C5—C6	2.0 (2)	C6—C5—C14—C13	−57.03 (14)
C3—C4—C5—C14	−176.46 (13)	C12—C13—C14—C15	13.99 (19)
C2—C1—C6—C5	1.9 (2)	C8—C13—C14—C15	−167.52 (11)
C2—C1—C6—C7	179.78 (14)	C12—C13—C14—C17	129.29 (14)
C4—C5—C6—C1	−3.4 (2)	C8—C13—C14—C17	−52.22 (14)
C14—C5—C6—C1	175.29 (12)	C12—C13—C14—C5	−117.89 (14)
C4—C5—C6—C7	178.45 (12)	C8—C13—C14—C5	60.60 (13)
C14—C5—C6—C7	−2.83 (17)	C16—O2—C15—O1	−179.23 (15)
C1—C6—C7—C8	−118.49 (16)	C16—O2—C15—C14	1.30 (19)
C5—C6—C7—C8	59.48 (15)	C17—C14—C15—O1	178.30 (16)
C1—C6—C7—C18	128.95 (16)	C5—C14—C15—O1	61.1 (2)
C5—C6—C7—C18	−53.07 (16)	C13—C14—C15—O1	−64.7 (2)
C18—C7—C8—C9	−125.80 (15)	C17—C14—C15—O2	−2.28 (16)
C6—C7—C8—C9	121.28 (15)	C5—C14—C15—O2	−119.45 (14)
C18—C7—C8—C13	57.06 (14)	C13—C14—C15—O2	114.75 (14)
C6—C7—C8—C13	−55.86 (15)	C15—O2—C16—C17	0.30 (19)
C13—C8—C9—C10	1.4 (2)	O2—C16—C17—C18	−178.90 (18)
C7—C8—C9—C10	−175.55 (13)	O2—C16—C17—C14	−1.73 (17)
C8—C9—C10—C11	0.0 (2)	C15—C14—C17—C18	−179.75 (13)
C9—C10—C11—C12	−1.2 (2)	C5—C14—C17—C18	−55.14 (16)
C10—C11—C12—C13	0.9 (2)	C13—C14—C17—C18	56.61 (16)
C11—C12—C13—C8	0.5 (2)	C15—C14—C17—C16	2.39 (16)
C11—C12—C13—C14	178.84 (12)	C5—C14—C17—C16	127.00 (13)
C9—C8—C13—C12	−1.7 (2)	C13—C14—C17—C16	−121.24 (13)
C7—C8—C13—C12	175.68 (11)	C16—C17—C18—C7	175.33 (18)
C9—C8—C13—C14	179.70 (11)	C14—C17—C18—C7	−1.69 (19)
C7—C8—C13—C14	−2.94 (15)	C8—C7—C18—C17	−54.71 (16)
C4—C5—C14—C15	−9.8 (2)	C6—C7—C18—C17	56.09 (17)
C6—C5—C14—C15	171.65 (12)		

### Hydrogen-bond geometry (Å, º)

Cg1 and Cg2 are the centroids of the C1–C6 and C8–C13 rings, respectively.

**Table:**

*D*—H···*A*	*D*—H	H···*A*	*D*···*A*	*D*—H···*A*
C7—H7···*Cg*1^i^	0.98	2.92	3.7835 (16)	148
C16—H16*B*···*Cg*1^ii^	0.97	2.76	3.650 (2)	153
C18—H18···*Cg*2^i^	0.93	2.84	3.3942 (16)	120
C3—H3···O1^iii^	0.93	2.64	3.519 (2)	157

Symmetry codes: (i) *x*, −*y*−1/2, *z*−1/2; (ii) −*x*+1, −*y*, −*z*+1; (iii) −*x*+1, −*y*, −*z*.
